# Efficacy and tolerability of rivastigmine patch therapy in patients with mild-to-moderate Alzheimer’s dementia associated with minimal and moderate ischemic white matter hyperintensities: A multicenter prospective open-label clinical trial

**DOI:** 10.1371/journal.pone.0182123

**Published:** 2017-08-07

**Authors:** Kyung Won Park, Eun-Joo Kim, Hyun Jeong Han, Yong S. Shim, Jae C. Kwon, Bon D. Ku, Kee Hyung Park, Hyon-Ah Yi, Kwang K. Kim, Dong Won Yang, Ho-Won Lee, Heeyoung Kang, Oh Dae Kwon, SangYun Kim, Jae-Hyeok Lee, Eun Joo Chung, Sang-Won Park, Mee Young Park, Bora Yoon, Byeong C. Kim, Sang Won Seo, Seong Hye Choi

**Affiliations:** 1 Department of Neurology, Cognitive Disorders and Dementia Center, Dong-A University College of Medicine and Institute of Convergence Bio-Health, Busan, South Korea; 2 Department of Neurology, Pusan National University Hospital and Biomedical Research Institute, Pusan National University School of Medicine, Busan, South Korea; 3 Department of Neurology, Seonam University College of Medicine, Myongji Hospital, Goyang, South Korea; 4 Department of Neurology, Holy Family Hospital, The Catholic University of Korea, School of Medicine, Bucheon, South Korea; 5 Department of Neurology, Changwon Fatima Hospital, Changwon, South Korea; 6 Department of Neurology, Catholic Kwandong University College of Medicine, Gangneung, South Korea; 7 Department of Neurology, Gachon University Gil Hospital, Incheon, South Korea; 8 Department of Neurology, Keimyung University College of Medicine, Daegu, South Korea; 9 Department of Neurology, Dongguk University College of Medicine, Seoul, South Korea; 10 Department of Neurology, The Catholic University of Korea, School of Medicine, Seoul, South Korea; 11 Department of Neurology, Kyungpook National University School of Medicine, Daegu, South Korea; 12 Department of Neurology, Gyeongsang National University College of Medicine, Jinju, South Korea; 13 Department of Neurology, Catholic University of Daegu School of Medicine, Daegu, South Korea; 14 Department of Neurology, Seoul National University College of Medicine, Seoul National University Bundang Hospital, Seongnam, South Korea; 15 Department of Neurology, Pusan National University Yangsan Hospital, Yangsan, South Korea; 16 Department of Neurology, Busan Paik Hospital, Inje University College of Medicine, Busan, South Korea; 17 Department of Neurology, Daegu Fatima Hospital, Daegu, South Korea; 18 Department of Neurology, Yeungnam University College of Medicine, Daegu, South Korea; 19 Department of Neurology, Konyang University College of Medicine, Daejeon, South Korea; 20 Department of Neurology, Chonnam National University School of Medicine, Gwangju, South Korea; 21 Department of Neurology, Samsung Medical Center, Sungkyunkwan University School of Medicine, Seoul, South Korea; 22 Department of Neurology, Inha University School of Medicine, Incheon, South Korea; University of Toronto, CANADA

## Abstract

**Background and objective:**

Studies investigating the impact of white matter hyperintensities (WMHs) on the response of acetylcholinesterase inhibitors in patients with Alzheimer’s disease (AD) have presented inconsistent results. We aimed to compare the effects of the rivastigmine patch between patients with AD with minimal WMHs and those with moderate WMHs.

**Methods:**

Three hundred patients with mild to moderate AD were enrolled in this multicenter prospective open-label study and divided into two groups. Group 1 comprised patients with AD with minimal WMHs and group 2 comprised those with moderate WMHs. The patients were treated with a rivastigmine patch for 24 weeks. Efficacy measures were obtained at baseline and after 24 weeks. The primary endpoint was the change in the AD Assessment Scale-Cognitive subscale (ADAS-Cog) from the baseline to the end of the study.

**Results:**

Of the 300 patients, there were 206 patients in group 1 and 94 patients in group 2. The intention-to-treat group comprised 198 patients (group 1, n = 136; group 2, n = 46) during the 24-week study period. Demographic factors did not differ between group 1 and group 2. There were no significant differences in change in ADAS-cog between group 1 (-0.62±5.70) and group 2 (-0.23±5.98) after the 24-week rivastigmine patch therapy (*p* = 0.378). The patients in group 1 had a 0.63-point improvement from baseline on the Frontal Assessment Battery, while group 2 had a 0.16-point decline compared to baseline at the end of the study (*p* = 0.037). The rates of adverse events (AEs) (42.6 vs. 40.3%) and discontinuation due to AEs (10.3% vs. 4.3%) did not differ between the groups.

**Conclusions:**

Although the efficacy and tolerability of rivastigmine patch therapy were not associated with WMH severity in patients with AD, some improvement in frontal function was observed in those with minimal WMHs.

**Trial registration:**

ClinicalTrials.gov NCT01380288

## Introduction

Alzheimer’s disease (AD) is the most common cause of neurodegenerative dementia [1]. Degenerative changes in cholinergic neurons of the nucleus basalis of Meynert, which provides the major cholinergic input to the cerebral cortex, hippocampus, and temporal cortex, lead to acetylcholine depletion. This depletion is associated with cognitive, behavioral, and functional impairments in AD [2]. The safety and efficacy of three acetylcholinesterase inhibitors (AChEIs)–donepezil, galantamine, and rivastigmine–in terms of cognitive improvement in AD have been confirmed in multicenter placebo-controlled double-blind randomized trials, and they have been widely used for the symptomatic relief of AD[3–5].

Vascular dementia (VaD), the second most common cause of dementia, accounts for about 20% of all dementia cases. Pure VaD is mainly caused by cerebrovascular disease (CVD) or small-vessel disease (SVD), but over 40% of VaD is often mixed with AD pathology [6]. Indeed, there is growing evidence that parallel cerebrovascular and neurodegenerative pathologies are observed in AD and VaD (mixed AD with cerebrovascular disease). Furthermore, it has been reported that several vascular risk factors play important roles in the development of AD [7, 8].

Since cholinergic structures, such as the basal forebrain and hippocampal CA1, are vulnerable to ischemic injury and widespread white matter bundles of both the lateral and medial cholinergic pathways reach almost all areas of the neocortex [9], cerebrovascular disease, such as localized stroke or microangiopathy mainly involving white matter may interrupt these cholinergic pathways[10]. Since AD and VaD share the common neurochemical characteristics of cortical cholinergic depletion, AChEIs have been used as the major treatment for pure VaD or mixed AD with CVD, as well as pure AD.

Rivastigmine, which inhibits both acetylcholinesterase and butyrylcholinesterase (BuChE), was previously shown to be beneficial in preventing neuronal degeneration by increasing regional cerebral blood flow in animal models [11]. The neuroprotective effects of rivastigmine in the context of ischemic brain conditions have also been observed in animal studies[12–14]. Thus, rivastigmine may be an important treatment option for AD with concurrent vascular pathology.

In fact, one previous randomized trial has indicated that following rivastigmine treatment for 26 weeks, patients with AD with vascular risk factors (VRFs) showed greater clinical benefit in cognition, activities of daily living, and disease severity than those with AD without VRFs [15]. Another recent retrospective analysis of a large international 24-week multicenter randomized double-blind placebo-and active-controlled trial also indicated the significant impact of VRF status on treatment response in AD [16]. In these studies, however, VRFs were determined using only the Modified Hachinski Ischemic Score (HIS) [17] or by assessing the presence or absence of reported VRFs at the time of screening. Therefore, it remains unknown whether the patients with AD with VRFs had actual concurrent CVD pathology as confirmed by brain magnetic resonance imaging (MRI). Furthermore, there have been no studies on the effects of rivastigmine patch in patients with AD with varying degrees of WMH. According to some studies [18, 19], the presence of WMH predicted a favorable clinical response to donepezil on tasks assessing frontal cognitive functions, while other studies reported the presence of WMH was associated with a poorer response to AChEIs [20] or that it did not influence the clinical response to AChEIs [21].

Thus, we sought to investigate the efficacy of the rivastigmine patch in patients with mild to moderate AD with minimal versus moderate ischemic WMHs. The treatment effect of rivastigmine is generally greater in patients with AD with VRF than in those with AD without VRF [15], and VRFs are associated with greater WMHs or reduced white matter integrity [22,23]. We thus hypothesized that patients with AD with moderate WMHs would have greater beneficial effects from baseline than those with AD with minimal WMHs. To maximize the therapeutic effects of rivastigmine on cognitive function, we used a transdermal 9.5 mg/24hour rivastigmine patch (10cm^2^), which offers continuous delivery of the drug with minimal plasma fluctuations. This delivery method provides to potentially comparable efficacy and fewer side effects compared to the highest dose of the rivastigmine capsule [24,25].

## Materials and methods

### Participants

Eligible patients were between 50 and 90 years of age and had a diagnosis of probable AD according to National Institute of Neurological and Communicative Disorders and Stroke, and the Alzheimer’s Disease and Related Disorders Association (NINCDS-ADRDA) [26]. All subjects had mild to moderately severe cognitive impairment as defined by Mini-Mental State Examination (MMSE) scores of 10–26 [27], minimal or moderate ischemia as indicated by an MRI scan conducted at baseline or within 12 months prior to the baseline examination based on the Clinical Research Center for Dementia of South Korea (CREODS) WMH visual rating scale [28], HIS≤ 4, and a responsible care provider who had contact with the patient at least once per week and could be a reliable informant. None of the patients had clinically significant laboratory abnormalities, such as thyroid disease, vitamin B12 deficiency or folic acid deficiency.

Patients were excluded from the study if they had received an investigational medication, AChEIs, N-methyl-D-aspartate receptor antagonist, or anticholinergic drugs within 4 weeks prior to the screening or if they had evidence of active skin lesions, allergy to the study drugs, or any advanced or unstable disease that would prevent completion of the study. Diseases precluding enrolment in the study included acute and severe asthmatic conditions, severe and unstable cardiovascular disease (bradycardia with heart rate < 50 bpm, sick sinus syndrome, sinoatrial block, or 2^nd^/3^rd^ degree atrioventricular block), active peptic ulceration or gastrointestinal bleeding, severe hepatic or renal disease, malignancy within the past 5 years, or severely declined vision or hearing. Other exclusion criteria included a history or presence of any contraindication for the application of AchEIs, a history of other concomitant neurodegenerative or psychiatric disease, a history of drug or alcohol addiction within the previous 10 years, or severe ischemia based on the CREDOS WMH visual rating scale, and multiple large territorial infarctions or single strategically placed infarctions on MRI scan conducted at baseline or within 12 months of the baseline examination.

The study was performed in accordance with the International Harmonization Conference guidelines on Good Clinical Practice and was approved by the Dong-A University Hospital Institutional Review Board (IRB) and IRB ofeach center prior to the beginning the study. Prior to participation in the study, all participants or their legally authorized representatives provided written informed consent to participate in the study. This study was registered at clinicaltrials.gov as NCT01380288.

### Study design

This was a 24-week prospective open-label multicenter trial conducted at 20 centers across South Korea between July 6, 2011 and December 8, 2014 (date of the last patient’s last visit). After assessments for eligibility performed over a 4-week screening period, patients underwent baseline efficacy and safety assessments and were divided into two groups based on their CREDOS WMH visual rating scale scores. In brief, this classification system was developed using a combination of deep WMH (DWMH) and periventricular WMH (PWMH) scores. On this scale, PWMHs were classified as P1 (cap or band <5 mm), P2 (≥5 mm cap or band < 10 mm), and P3 (cap or band ≥ 10 mm), while DWMH were classified as D1 (maximum diameter of deep white matter lesion < 10 mm), D2 (10 mm ≤ lesion <25 mm), and D3 (≥25 mm). Combinations of D1 and P1 (D1P1) and D1 and P2 (D1P2) were classified as “minimal.” The D1P3, D2P1, D2P2, D2P3, D3P1, and D3P2 combinations were classified as “moderate,” and D3P3 was classified as “severe” [28]. Group 1 included patients with AD with minimal WMHs (D1P1 and D1P2), and group 2 included patients with AD with moderate WMHs (D1P3, D2P1, D2P2, D2P3, D3P1, and D3P2). All MRIs of participants were examined by 3 experienced neurologists blind to clinical information within the screening period and the WMH visual rating scales were assigned by consensus.

Participants in both groups were initially treated using on a 4.6mg/24hours rivastigmine patch (5cm^2^) and were then up-titrated to a 9.5mg/24hours rivastigmine patch (10cm^2^) over 4weeks. This was followed by a 20-week maintenance phase. If patients were unable to reach the target dose during the titration period due to tolerability problems, the dose was increased over 8 weeks.

### Outcomes

Efficacy measures were assessed at baseline and at week 24. The primary outcome was the change from baseline on the Alzheimer’s Disease Assessment Scale-Cognitive subscale at week 24 (ADAS-Cog) [29,30]. Secondary outcomes were as follows: MMSE, Clinical Dementia Rating Scale-Sum of Boxes (CDR-SB) [31], Frontal Assessment Battery (FAB) [32], Caregiver-Administered Neuropsychiatric Inventory (CGA-NPI) [33], Alzheimer’s Disease Cooperative Study-Activities of Daily Living (ADCS-ADL) [34], and Caregiver Burden Scale-Korean Version of Mini-Zarit [35]. Safety evaluations, including vital signs, neurological examinations, and adverse event (AE) and serious AE (SAE) monitoring were performed regularly (baseline, 4^th^ week, 12^th^ week and 24^th^ week) for both groups throughout the study.

### Sample size

The sample size calculation was based on the change from baseline to week 24 of the primary efficacy variable, the ADAS-Cog. A significant difference in the ADAS-Cog scores at 24 weeks between the two groups of patients (AD associated with minimal WMH, group 1; and AD with moderate WMH, group 2), was defined as a mean difference in scores of 2.3 points, which would be assessed using an independent t test at a significance level of 0.05 with a standard deviation (SD) of 6. Based on the above assumption, 162 participants for group 1 and 81 participants for group 2 were required. Taking into account an expected dropout rate of 20%, a total of 300 patients, with 200 in group 1 and 100 in group 2, were sought for recruitment.

### Statistical analyses

Data were summarized using descriptive statistics: frequency and percentage for categorical variables and mean ± SD for continuous variables. Differences in study participants' characteristics were compared across subgroups using chi-square tests or Fisher’s exact tests for categorical variables, and independent t tests or Mann-Whitney’s U tests for continuous variables, as appropriate. To check for normal distribution, we used the Shapiro-Wilk test. Changes from baseline to end point were compared using an analysis of covariance (ANCOVA) model, using the baseline score as a covariate. We used bar charts for data visualization. Intent to treat (ITT) and per protocol (PP) analyses were performed and the last observed carried forward (LOCF) method was used to impute missing values. *P*-values less than 0.05 were considered statistically significant. All statistical analyses were carried out using Statistical Package for Social Sciences 22.0 (SPSS Statistics for Windows 22.0, Armonk, NY, IBM Corp.) software. All tests were two-tailed.

## Results

### Demographics

**Fig 1:**
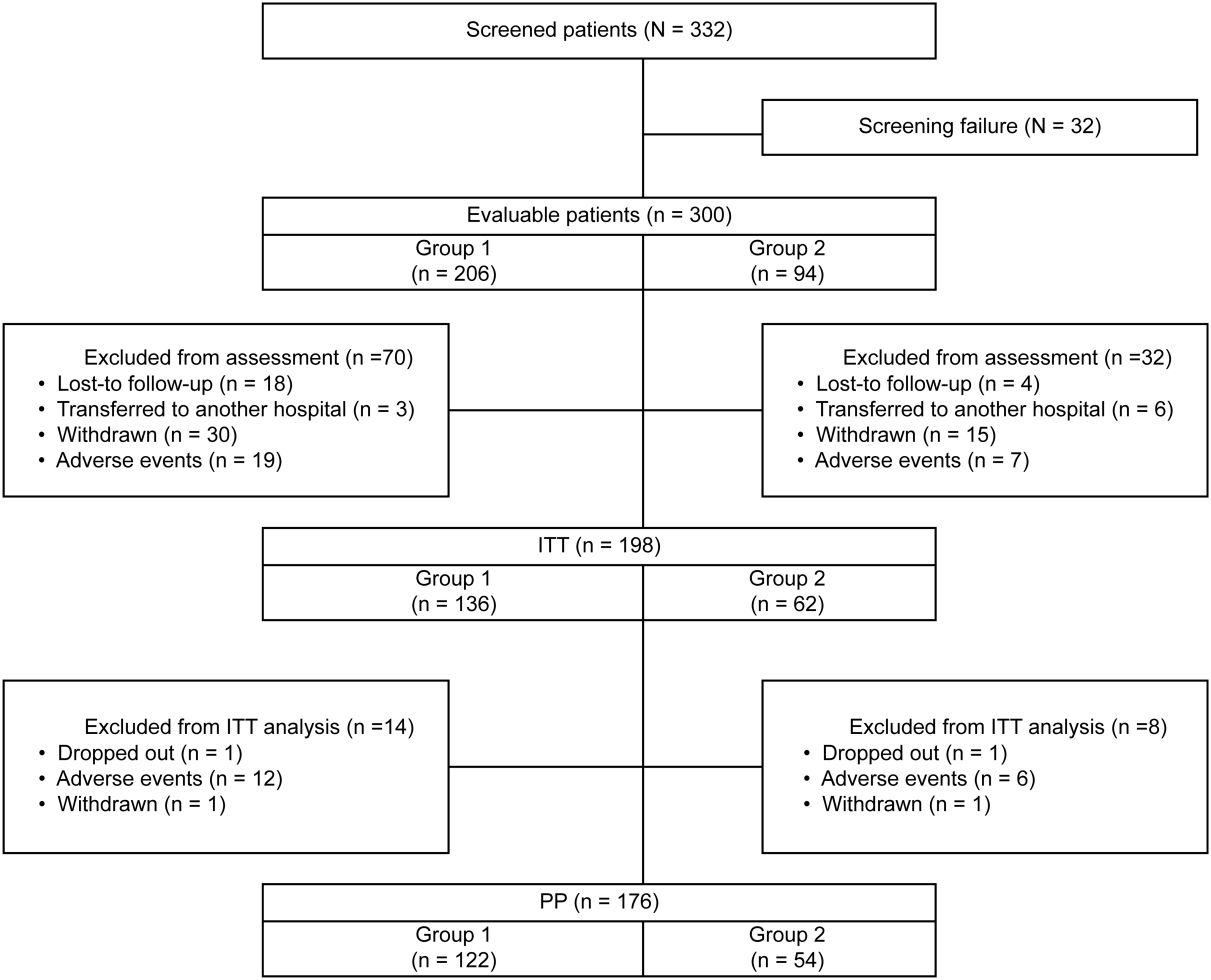
Flow chart of the study design and patient participation.

A total of 332 patients were screened for inclusion and 32 failed the screening. Of the 300 patients enrolled in the study, 206 had minimal WMH (group 1) and 94 had moderate WMH (group 2). Seventy patients in group 1 and 32 patients in group 2 were excluded from the efficacy assessment due to loss to follow-up (n = 22), transfer to another hospital (n = 9), withdrawal (n = 45), or AEs (n = 26). Thus, the ITT population comprised 198 patients (group 1: n = 136, group 2: n = 62). There were no significant differences in demographics and baseline characteristics between group 1 and group 2 (Table 1). The PP populations consisted of 176 patients (122 in group 1 and 54 in group 2) from the ITT population for whom no major protocol violations (such as drop–out or AEs) were reported (Fig 1).

**Table 1:** Demographics and clinical baseline characteristics of the patients (ITT population, n = 198).

Variable	Group 1 (n = 136)	Group 2 (n = 62)	p-value
Age, years	73.35±8.08	75.61±6.34	0.085^1^
Gender			
Male	38 (27.9%)	20 (32.3%)	0.614^2^
Female	98 (72.1%)	42 (67.7%)	
Education, years	6.80±4.36	6.63±5.26	0.530^1^
Concurrent medical conditions			
Hypertension	57 (41.9%)	34 (54.8%)	0.091^2^
Diabetes mellitus	34 (25.0%)	20 (32.3%)	0.288^2^
Hyperlipidemia	23 (16.9%)	9 (14.5%)	0.671^2^
Heart Disease	15 (11.0%)	10 (16.1%)	0.316^2^
Depression	7 (5.1%)	4 (6.5%)	0.743^3^
Other disease	42 (30.9%)	22 (35.5%)	0.521^2^
ADAS-Cog	22.73±7.84	23.40±7.27	0.478^1^
MMSE	19.63±4.03	19.95±4.10	0.550^1^
FAB	10.15±3.47	10.08±3.44	0.997^1^
ADCS-ADL	59.32±12.39	59.24±11.97	0.866^1^
CGA-NPI	10.51±12.57	8.89±11.48	0.202^1^
CDR-SB	3.89±2.21	4.17±2.26	0.308^1^
Mini-Zarit (caregiver burden)	18.93±14.83	17.79±13.36	0.699^1^

^1^P values were derived from the Mann-Whitney U test;

^2^P values were derived from the chi-square test;

^3^P values were derived from Fisher’s exact test. The Shapiro-Wilk test was used to test for normality assumption.

Values are means ± standard deviations. ITT = intent to treat; LOCF = Last Observation Carried Forward; ADAS-Cog = Alzheimer’s Disease Assessment Scale-Cognitive subscale; MMSE = Mini-Mental State Examination; FAB = Frontal Assessment Battery; CGA-NPI = Caregiver-Administered Neuropsychiatric Inventory; ADSC-ADL = Alzheimer’s Disease Cooperative Study-Activities of Daily Living; CDR-SB = Clinical Dementia Rating Scale-Sum of Boxes.

### Efficacy

**Fig 2:**
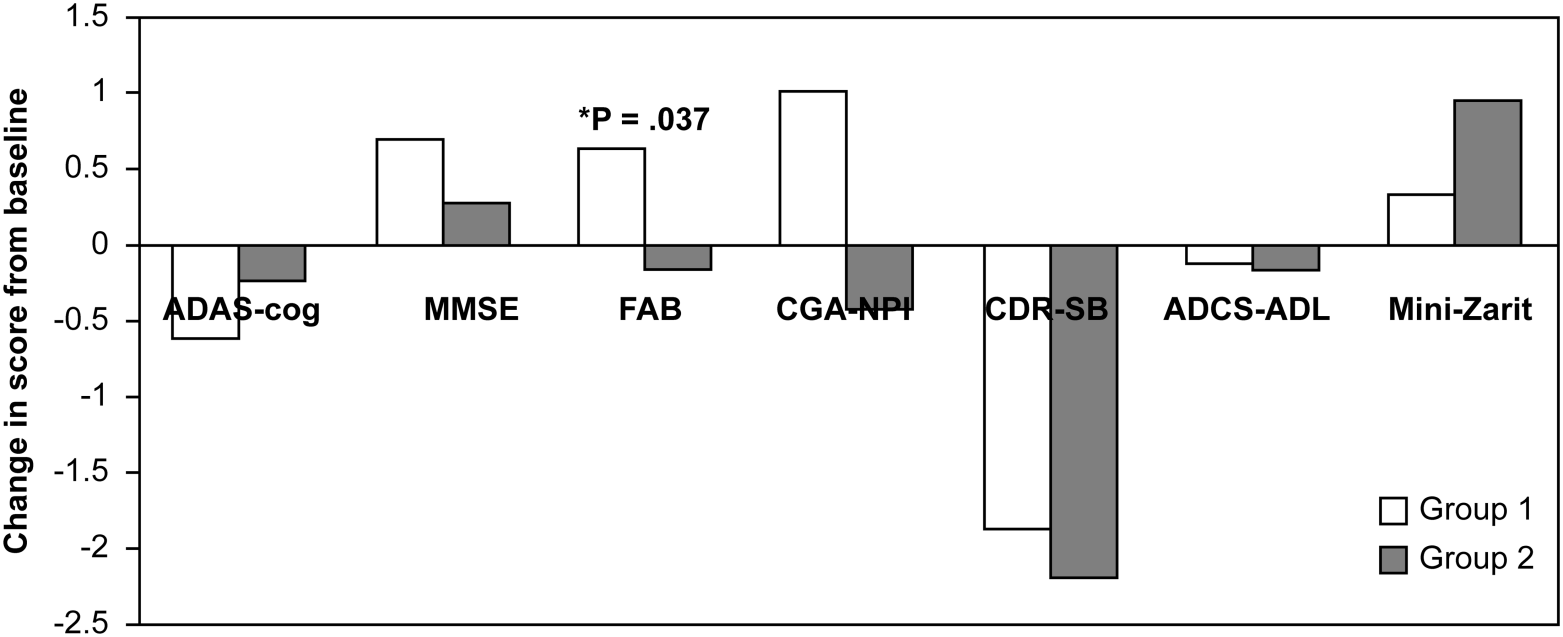
Changes from the baseline at 24 weeks in the ADAS-cog, MMSE, FAB, CGA-NPI, CDR-SB, ADCS-ADL, and Mini-Zarit (ITT-LOCF population) in the patients from groups 1 and 2.

No significant differences in the change from baseline to week 24 on the ADAS-Cog between group 1 and group 2 were observed in the ITT-LOCF (p = 0.378) and PP populations (p = 0.442). Table 2 shows the mean change in cognition, behavior, ADL, and caregiver’s burden in both groups at 24 weeks. Significant differences between group 1 and group 2 were observed only in FAB scores (p = 0.037) (Fig 2). Group 1 had an improvement of 0.6 points in the FAB score over baseline, while group 2 experienced a decline to 0.2 points below baseline.

**Table 2:** Efficacy outcomes at week 24 (observed cases) and at endpoint (ITT-LOCF population).

	Baseline score		Change from baseline					
	ITT population		ITT-LOCF population			PP population (week 24 observed cases)		
	Group 1 (n = 136)	Group 2 (n = 62)	Group 1 (n = 136)	Group 2 (n = 62)	*p*	Group 1 (n = 122)	Group 2 (n = 54)	*p*
ADAS-Cog	22.73 ± 7.84	23.40 ± 7.27	-0.62 ± 5.70	-0.23 ± 5.98	0.378	-0.58 ± 5.57	-0.13 ± 6.17	0.442
MMSE	19.63 ± 4.03	19.95 ± 4.10	0.70 ± 2.70	0.26 ± 2.87	0.298	0.66 ± 2.81	0.17 ± 2.99	0.290
FAB	10.15 ± 3.47	10.08 ± 3.44	0.63 ± 2.80	-0.16 ± 2.62	**0.037**	0.62 ± 2.79	-0.04 ± 2.58	0.116
CGA-NPI	59.32 ± 12.39	59.24 ± 11.97	1.01 ± 8.89	-0.44 ± 10.09	0.178	1.23 ± 9.24	-0.48 ± 10.62	0.167
CDR-SB	10.51 ± 12.57	8.89 ± 11.48	-1.89 ± 10.64	-2.21 ± 8.49	0.804	-2.26 ± 10.76	-1.91 ± 8.62	0.445
ADCS-ADL	3.89 ± 2.21	4.17 ± 2.26	-0.13 ± 0.92	-0.15 ± 1.47	0.544	-0.07 ± 0.91	-0.20 ± 1.52	0.805
Mini-Zarit	18.93 ± 14.83	17.79 ± 13.36	0.34 ± 12.46	0.95 ± 12.39	0.613	0.83 ± 12.21	1.19 ± 12.15	0.688

Values are mean±standard deviation. ITT = intent-to-treat; LOCF = Last Observation Carried Forward; PP = per protocol; ADAS-Cog = Alzheimer’s Disease Assessment Scale-Cognitive subscale; MMSE = Mini-Mental State Examination; FAB = Frontal Assessment Battery; CGA-NPI = Caregiver-Administered Neuropsychiatric Inventory; ADSC-ADL = Alzheimer’s Disease Cooperative Study-Activities of Daily Living; CDR-SB = Clinical Dementia Rating Scale-Sum of Boxes.

The proportions of patients with MMSE scores under 20 with equal or better scores in efficacy variables at the end of the treatment period compared to baseline are shown in Table 3. The responder rates for the FAB had a nearly significant tendency to be higher in group 1 compared to those in group 2 (69.4% vs. 48.1%, *p* = 0.057; Table 3). There were no significant differences between the groups in the responder rates for the other efficacy variables. The comparisons of responder rates at week 24 in patients with MMSE ≥ 20 were also performed, however, no significant differences were observed between the groups in all efficacy variables (data were not shown).

**Table 3:** Comparisons of responder rates at week 24 in patients with MMSE < 20.

Variable	Group 1 (n = 62)	Group 2 (n = 27)	*p*-value*
ADAS-Cog (Δ≤0)	42 (67.7%)	16 (59.3%)	0.440
MMSE (Δ≥0)	46 (74.2%)	20 (74.1%)	0.991
FAB (Δ≥0)	43 (69.4%)	13 (48.1%)	0.057
ADCS-ADL (Δ≥0)	40 (64.5%)	16 (59.3%)	0.637
CGA-NPI (Δ≤0)	41 (66.1%)	20 (74.1%)	0.458
CDR-SB (Δ≤0)	47 (75.8%)	21 (77.8%)	0.840
Mini-Zarit(Δ≤0)	31 (50.0%)	11 (40.7%)	0.421

*P values were derived from the chi square test.

### Safety

The number and percentage of patients who experienced AEs are summarized in Table 4. Itching (17.2%) and rash (11.6%) were the most commonly reported AEs. A total of 15 SAEs, including one death, were reported. However, there were no SAEs associated with the study drug (Table 4). There were no significant differences in the incidence of AEs and SAEs between the groups. Forty-four patients with AEs (including 1 patient with an SAE) permanently discontinued the study. There was no significant difference in the incidence of discontinuation between group 1 (n = 31, 10.3%) and group 2 (n = 13, 4.3%). Skin irritation was the most common cause of discontinuation (n = 36, 12%).

**Table 4:** All adverse events in both treatment groups.

	Overall (n = 198)	Group 1 (n = 136)	Group 2 (n = 62)	*p*-value*
Adverse events (AEs)				
Itching	34 (17.2%)	21 (15.4%)	13 (21.0%)	0.339
Rash	23 (11.6%)	16 (11.8%)	7 (11.3%)	0.923
Nausea	10 (5.1%)	8 (5.9%)	2 (3.2%)	0.728
Vomiting	6 (3.0%)	5 (3.7%)	1 (1.6%)	0.667
Headache	5 (2.5%)	3 (2.2%)	2 (3.2%)	0.649
Skin eruption	6 (3.0%)	6 (4.4%)	0 (0.0%)	0.180
Dizziness	2 (1.0%)	2 (1.5%)	0 (0.0%)	1.000
Anorexia	1 (0.5%)	1 (0.7%)	0 (0.0%)	1.000
Dyspnea	1 (0.5%)	0 (0.0%)	1 (1.6%)	0.313
Edema	1 (0.5%)	1 (0.7%)	0 (0.0%)	1.000
Epigastric discomfort	1 (0.5%)	1 (0.7%)	0 (0.0%)	1.000
Epigastric pain	1 (0.5%)	0 (0.0%)	1 (1.6%)	0.313
Fracture	1 (0.5%)	0 (0.0%)	1 (3.2%)	0.313
Paresthesia	1 (0.5%)	1 (0.7%)	0 (0.0%)	1.000
Shoulder pain	1 (0.5%)	1 (0.7%)	0 (0.0%)	1.000
Upper respiratory infection	1 (0.5%)	1 (0.7%)	0 (0.0%)	1.000
Serious Adverse events (SAEs)				
Abscess	1 (0.5%)	1 (0.7%)	0 (0.0%)	1.000
Fracture	1 (0.5%)	0 (0.0%)	1 (1.6%)	0.313
Bradycardia	1 (0.5%)	1 (0.7%)	0 (0.0%)	1.000
Zoster	1 (0.5%)	1 (0.7%)	0 (0.0%)	1.000

* *P*-values were derived from the chi square test.

## Discussion

Our results indicate that patients with AD with minimal WMHs had better responses to the rivastigmine patch compared to those of the patients with AD with moderate WMHs in terms of frontal lobe function, which was assessed using the FAB score. No significant differences in the changes in the primary outcome measure of ADAS-cog or other secondary efficacy measures between patients with AD with minimal WMHs and those with moderate WMHs were observed after 24 weeks of rivastigmine patch therapy.

One possible explanation for these findings might be that in AD with moderate WMHs, the degree of cholinergic depletion due to ischemic damage to the cholinergic pathway maybe greater than that in AD with minimal WMHs. This may reduce the efficacy of AChEI treatment. This may also explain the results of previous studies showing that cerebral metabolic changes in response to cognitive or psychophysical stimulation are normal during the early stages and are delayed in the later stages of AD. In other words, the effects of AChEIs may be attenuated when the hyperintensities involve more cholinergic pathways according to disease progression [18, 36]. Thus, higher reserves of acetylcholine in the frontal areas may lead to more efficient responses to AChEI treatment.

Several studies have investigated the influence of WMHs on the response to AChEIs in AD, VaD, and AD with CVD, although their results have been inconsistent [18–21, 37]. Erkinjuntti et al. reported that patients with AD combined with CVD treated with galantamine 24 mg/day for 6 months showed significant improvements in cognition and global functioning compared to those treated with placebo [37]. Serial studies from Japan suggest that WMH might be an MRI parameter predicting a favorable response to donepezil in terms of frontal function, but not in terms of memory or language function [18,19]. Connelly et al. reported, however, that patients with AD with combined HT and WMH had poorer responses to AChEIs than patients with either HT or WMH as well as those with neither condition [20]. Devine et al. also reported that WMH severity visually rated on MRI or computed tomography did not influence the clinical response to AChEIs in patients with AD [21]. Various methods used to measure the severity of WMH and different follow-up durations may explain the inconsistent results of those studies.

WMH in AD is known to be associated with executive dysfunction and psychomotor slowing. It has been suggested that deficits in executive function caused by WMH are more responsive to AChEIs than are other cognitive dysfunctions [38]. In our study, in line with this proven rationale, patients with AD with minimal WMHs improved in terms of frontal function. These findings are in agreement with the findings of other studies on rivastigmine, wherein significant bilateral increases in frontal, temporal, and parietal brain perfusion, enhanced frontal activation, or bilateral increases in regional frontal cerebral blood flow were observed in patients with AD or VaD [39–41]. In addition, a study showing the selective affinity of rivastigmine for frontal areas may explain the observed beneficial effects of this drug on frontal function in our study [42].

Although several open-label clinical trials of rivastigmine conducted in patients with VaD have demonstrated significantly improved behavior and executive function[43–45], a large-scale randomized double-blind placebo-controlled clinical trial of rivastigmine in patients with probable VaD showed unsatisfactory results[46]. VaD is heterogeneous and the damage to cholinergic pathways due to vascular injury is difficult to clearly define. Therefore, despite the theoretical rationales for treatment of VaD with AChEIs discussed in the introduction such studies may result in incoherent outcomes.

The most frequent AEs were skin irritations, such as itching and rash. Compared to a previous western study, the incidence of skin reaction associated with the rivastigmine patch was somewhat higher. However, gastrointestinal AEs in our study were comparable to those in a western study [47]. When compared to other Asian studies, the incidence of skin reaction associated with the rivastigmine patch was lower and gastrointestinal AEs occurred less frequently in our study [30,48]. No drug-drug interactions or SAEs related to the study drug were reported. Thus, as suggested in other studies of transdermal rivastigmine patch, our results support the viability of the rivastigmine patch as a treatment option for older patients with AD who are taking many other concomitant medications.

This study has a few limitations. First, this is an open-label study without a placebo group, and with a relatively high dropout rate. Second, WMH was measured in a semiquantitative manner. Third, diagnoses of AD with minimal or moderate WMH were not confirmed by neuropathological or biomarker measurements. Nevertheless, our data suggest that when considering treatment options for patients with AD with WMH, AChEIs may confer more benefits for patients with AD with minimal WMHs than those with moderate WMHs. Our study results suggest that patients with milder WMHs have more AD pathology and therefore a higher cholinergic deficit, and those patients with moderate WMHs have less AD pathology with more VaD pathology and are less likely to have benefit from rivastigmine therapy. Further studies with placebo controls, longer time periods, and quantitatively measured WMH are needed to establish the efficacy of rivastigmine in AD with minimal or moderate WMH.

## Conclusion

There were no significant differences in general cognitive function between patients with AD with minimal vs. moderate white matter hyperintensities after 24 weeks of rivastigmine patch therapy. However, a significant improvement in frontal function was found in patients with AD with minimal WMHs following rivastigmine patch therapy compared to patients with AD with moderate WMHs.

## Supporting information

S1 Dataset Dataset for this manuscript. (XLSX)

S1 Protocol Study protocol for this manuscript. (DOC)

S1 Checklist TREND statement checklist. (PDF)

S2 Checklist TREND statement checklist. (PDF)

S3 Checklist TREND statement checklist. (PDF)
