# Supplementary material for: Efficacy and tolerability of rivastigmine patch therapy in patients with mild-to-moderate Alzheimer’s dementia associated with minimal and moderate ischemic white matter hyperintensities: A multicenter prospective open-label clinical trial
Source: PLoS One. 2017 Aug 7;12(8):e0182123. doi: 10.1371/journal.pone.0182123 (PMC5546604; doi:10.1371/journal.pone.0182123)
Supplement: S1 Protocol — (DOC) [file pone.0182123.s002.doc]

| **Protocol of Clinical Trial** |
| --- |

**Study Title:** Changes of cognitive function in patients with mild to moderate Alzheimer’s disease associated with or without white matter lesions after RivastigminE patch thERapy – A multi-center, prospective, open-label clinical trial (CAREER study)

NCC code:CENA713DKR11T

Protocol No: CAREER-001

Version 1.7

Investigator Initiated Trial

2014-01-21

**Table of contents**

1. Study title and design 1

[1.1 Study title](#__RefHeading___Toc199745552) 1

[1.2 Study design](#__RefHeading___Toc199745553) 1

[2. Principle investigator and co-investigators](#__RefHeading___Toc199745554) 1

[3. Background](#__RefHeading___Toc199745557) 3

[4. Objectives](#__RefHeading___Toc199745557) 4

[4.1 Rationale of the study](#__RefHeading___Toc199745559) 4

[4.2 Primary objective](#__RefHeading___Toc199745559) 5

[4.3 Secondary objective](#__RefHeading___Toc199745559) 5

[5. Study population](#__RefHeading___Toc199745560) 5

[5.1 Study population](#__RefHeading___Toc199745562) 5

[5.2 Number of subjects and centers](#__RefHeading___Toc199745563) 5

[5.3 Sample size justification](#__RefHeading___Toc199745564) 6

[5.4 Inclusion criteria](#__RefHeading___Toc199745565) 6

[5.5 Exclusion criteria](#__RefHeading___Toc199745566) 7

[6. Intervention](#__RefHeading___Toc199745569) 7

[6.1 Investigational drug](#__RefHeading___Toc199745562) 7

[6.2 Treatment regimen](#__RefHeading___Toc199745564) 7

[6.2.1 Treatment schedule](#__RefHeading___Toc199745565) 7

[6.2.2 Subject assignment and prescribing and administering medication](#__RefHeading___Toc199745566) 8

[6.2.3 Controlling medication doses and suspending doses](#__RefHeading___Toc199745566) 9

[6.2.4 Concomittent medication](#__RefHeading___Toc199745565) 9

[6.2.5 Discontinuation of trial drug treatment and early elimination of subjects](#__RefHeading___Toc199745566) 10

[6.2.6 Termination of study](#__RefHeading___Toc199745565) 11

[6.2.7 Early termination of study](#__RefHeading___Toc199745566) 11

[7. Study visit and assessment](#__RefHeading___Toc199745570) 12

[7.1 Subject demographic and background information](#__RefHeading___Toc199745562) 14

[7.2 Treatment exposure and compliance](#__RefHeading___Toc199745563) 14

[7.3 Efficacy](#__RefHeading___Toc199745564) 14

[7.4 Safety](#__RefHeading___Toc199745562) 15

[7.4.1 Physical and neurologic examination](#__RefHeading___Toc199745565) 15

[7.4.2 Height, weight, vital signs](#__RefHeading___Toc199745566) 15

[7.4.3 Adverse events](#__RefHeading___Toc199745566) 15

[7.5 Report of serious adverse events](#__RefHeading___Toc199745563) 17

[7.6 Report of serious adverse events](#__RefHeading___Toc199745563) 17

[8. Data analysis](#__RefHeading___Toc199745571) 20

[8.1 Analysis group](#__RefHeading___Toc199745562) 20

[8.2 Subject demographic and background information](#__RefHeading___Toc199745563) 20

[8.3 Analysis of primary objective](#__RefHeading___Toc199745563) 20

[8.3.1 Evaluation variables Statistical hypothesis, model and method analysis](#__RefHeading___Toc199745565) 20

[8.4 Analysis of secondary objective](#__RefHeading___Toc199745564) 21

[9. Ethical consideration](#__RefHeading___Toc199745572) 21

[9.1 Compliance with related laws and ethical principles](#__RefHeading___Toc199745562) 21

[9.2 Subject consent procedure](#__RefHeading___Toc199745563) 21

[9.3 Compliance with clinical trial plan](#__RefHeading___Toc199745564) 21

[9.4 Changes in clinical trial plan](#__RefHeading___Toc199745563) 21

[10. References](#__RefHeading___Toc199745573) 22

**1. Study title and design**

**1.1 Study title**

Title: **C**hanges of cognitive function in patients with mild to moderate **A**lzheimer’s disease associated with or without white matter lesions after **R**ivastigmin**E** patch th**ER**apy – multi-center, prospective, open-label clinical trial (**CAREER study**)

**1.2 Study design**

Investigator Initiative Trial, Multi-center, Prospective, Open-label

**2. Principle investigator and co-investigators**

|  | Institute | Department | Name | |
| --- | --- | --- | --- | --- |
| **Principle Investigator** | Dong-A University Hospital | Neurology | Kyung Won Park | |
|  | Pusan National University Hospital | Neurology | | Eun-Joo Kim |
|  | Myongji Hospital | Neurology | | Hyun Jeong Han |
|  | Holy Family Hospital | Neurology | | Yong S. Shim |
|  | Changwon Fatima Hospital | Neurology | | Jae C. Kwon |
|  | Catholic Kwandong University College of Medicine | Neurology | | Bon D. Ku |
|  | Gachon University Gil Hospital | Neurology | | Kee Hyung Park |
|  | Keimyung University College of Medicine | Neurology | | Hyon-Ah Yi |
|  | Dongguk University College of Medicine | Neurology | | Kwang K. Kim |
|  | The Catholic University of Korea | Neurology | | Dong Won Yang |
|  | Kyungpook National University School of Medicine | Neurology | | Ho-Won Lee |
|  | Gyeongsang National University College of Medicine | Neurology | | Heeyoung Kang |
|  | Catholic University of Daegu School of Medicine | Neurology | | Oh Dae Kwon |
|  | Seoul National University College of Medicine | Neurology | | SangYun Kim |
|  | Pusan National University Yangsan Hospital | Neurology | | Jae-Hyeok Lee |
|  | Busan Paik Hospital | Neurology | | Eun Joo Chung |
|  | Daegu Fatima Hospital | Neurology | | Sang-Won Park |
|  | Yeungnam University College of Medicine | Neurology | | Mee Young Park |
|  | Konyang University College of Medicine | Neurology | | Bora Yoon |
|  | Chonnam National University School of Medicine | Neurology | | Byeong C. Kim |
|  | Samsung Medical Center | Neurology | | Sang Won Seo |
|  | Inha University School of Medicine | Neurology | | Seong Hye Choi |

3. Study Background

Alzheimer’s disease (AD) is the most common cause of neurodegenerative dementia. Degenerative changes in cholinergic neurons of the nucleus basalis of Meynert, which provides the major cholinergic input to the cerebral cortex, hippocampus, and temporal cortex, lead to acetylcholine depletion. This is associated with cognitive, behavioral, and functional impairments in AD. The efficacy on cognitive improvement and safety of three acetylcholinesterase inhibitors (AChEIs)-donepezil, galantamine, and rivastigmine–in AD have been confirmed in multicenter placebo-controlled double-blind randomized trials, and they have been widely used for the symptomatic relief of AD.

Vascular dementia (VaD), which is the second most common cause of dementia, accounts for about 20% of all dementia cases. Pure VaD is mainly caused by cerebrovascular disease (CVD) or small-vessel disease (SVD), but over 40% of VaD is often mixed with AD pathology [6]. In other words, there is growing evidence that parallel cerebrovascular and neurodegenerative pathology is observed in AD and VaD (mixed AD with cerebrovascular disease). Furthermore, it has been reported that several vascular risk factors play important roles in the development of AD.

Since cholinergic structures, such as the basal forebrain and hippocampal CA1, are vulnerable to ischemic injury and widespread white matter bundles of both lateral and medial cholinergic pathways reach almost all areas of the neocortex, cerebrovascular disease, such as localized stroke or microangiopathy mainly involving white matter may interrupt these cholinergic pathways. Since AD and VaD share the common neurochemical characteristics of cortical cholinergic depletion, AChEIs have been used as the major treatment for pure VaD or mixed AD with CVD, as well as pure AD.

Rivastigmine, which inhibits both acetylcholinesterase and butyrylcholinesterase (BuChE), has previously been shown to be beneficial in preventing neuronal degeneration by increasing regional cerebral blood flowing both AD and VaD. The neuroprotective effects of rivastigmine in the context of ischemic brain conditions have also been observed in animal studies. Thus, rivastigmine may be an important treatment option for AD with concurrent vascular pathology.

In fact, one previous randomized trial has indicated that after rivastigmine treatment for 26 weeks, patients with AD with vascular risk factors (VRFs) had greater clinical benefit in cognition, activities of daily living, and disease severity than those with AD without VRFs. Another recent retrospective analysis of a large international 24-week multicenter randomized double-blind placebo-and active-controlled trial also indicates that VRF status seems to have a great impact on treatment response in AD. In these studies, however, VRFs were determined only using the Modified Hachinski Ischemic Score (HIS) or by assessing the presence or absence of reported VRFs at screening. It thus remains unknown whether patients with AD with VRFs had actual concurrent CVD pathology as assessed using brain magnetic resonance imaging (MRI). Furthermore, there have been no studies on the effects of rivastigmine patch in patients with AD with different degrees of WMH.

We thus investigated the efficacy of rivastigmine patch in patients with mild to moderate AD with minimal versus moderate ischemic WMHs. The treatment effect of rivastigmine is generally larger in patients with AD with VRF than in those with AD without VRF [15], and that VRFs are associated with greater WMHs or reduced white matter integrity [18,19]. We thus hypothesized that patients with AD with moderate WMHs will have greater clinical improvements from baseline than those with AD with minimal WMHs. To maximize the therapeutic effects of rivastigmine on cognitive function, we used a transdermal 9.5 mg/24hour rivastigmine patch (10cm^2^), which offers continuous delivery of the drug with minimal plasma fluctuations. This delivery method leads to potentially similar efficacy and fewer side effects compared to the highest dose of rivastigmine capsule.

**4. Objectives**

**4.1 Rationale of the study**

We hypothesized that rivastigmine patch will provide benefits to AD patients with white matter changes compared to those without any white matter changes. Possible explanation about favorable benefits for AD with white matter changes is that rivastigmine may act on both Alzheimer’s and vascular pathologies contributing to dementia, providing additive treatment effects in patients suffering from both conditions concurrently. To our Knowledge, there was no study or clinical trial to compare the changes of cognitive function, ADL, BPSD and caregiver burden in two groups of patient with Alzheimer’s disease associated with or without white matter changes after rivastigmine transdermal patch therapy.

**4.2 Primary objective**

To evaluate and compare the changes of cognitive function (as measured by ADAS-Cog) in the two group of patients with Alzheimer’s disease associated with and without white matter changes after rivastigmine patch therapy

**4.3 Secondary objective**

- To evaluate the change from baseline to week 24 of the following parameters in the two groups of patients with Alzheimer’s disease associated with and without white matter changes after rivastigmine patch therapy

- - MMSE (Mini-Mental State Examination)
  - Frontal Assessment Battery (FAB)
  - Alzheimer’s Disease Cooperative Study - Activities of Daily Living (ADCS-ADL)
  - Caregiver-Administered Neuropsychiatric Inventory (CGA-NPI)
  - Clinical Dementia Rating Scale-Sum of Boxes (CDR-SB)
  - Caregiver burden scale – Korean version of mini- Zarit
  - Adverse events

**5. Study population**

**5.1 Study population**

Probable Alzheimer’s dementia patients

**5.2 Number of subjects**

Three hundred patients

**5.3. Sample size justification**

In order to detect the difference in means of 2.3 (assumed from Kumar et al, 2000 as the difference between patients treated with 6-12 mg/day rivastigmine with and without vascular risk factors) of ADAS-cog change from baseline to Week 24 between AD patients with WMC and AD patients without WMC when a two-group t-test with a 0.05 two-sided significance level is used, the sample sizes in the two groups are 81 in AD patients with WMC and 162 for AD patients without WMC (a total sample size of 243), assuming that the common standard deviation is 6.0 (Winblad et al, 2007; Erkinjuntiti et al, 2002) and the allocation ratio of 1:2. The method of sample size calculation is as follows:


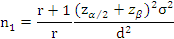
,

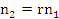


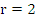


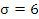


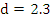


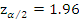


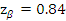


When the drop-out rate is expected to be 20%, a total number of required will be **300** patients (**100** in AD with WMC and **200** in AD without WMC).

**5. 4 Inclusion criteria**

1. Age range : 50 – 90 years of age
2. Sex distribution: both men and women. Females of non-childbearing potential or post-menopausal for at least 2 years
3. AD in NINCDS-ADRDA criteria, mild to moderate
4. probable AD with or without mild to moderate whiter matter lesions, excluding multiple large vessel infarcts or a single, strategically placed infarct (angular gyrus, thalamus, basal forebrain, territory of the posterior or anterior cerebral artery) on MRI scan (within 12 months)
5. MMSE score : 10 to 24 at screening
6. Hachinski scores ≤ 4
7. No clinically significant laboratory abnormalities, such as thyroid disease, vitamine B12 deficiency or folic acid deficiency

**5. 5 Exclusion criteria**

Patients were excluded from the study

1. Subjects who had received an investigational medication, AchEIs, N-methyl-D-aspartate receptor antagonist, or anticholinergic drugs within 4 weeks prior to screening or
2. Subjects who had evidence of active skin lesions, allergy to the study drugs, or any advanced or unstable disease that would prevent completion of the study.
3. Subjects who had diseases precluding enrolment in the study included acute and severe asthmatic conditions, severe and unstable cardiovascular disease (bradycardia with heart rate < 50 bpm, sick sinus syndrome, sinoatrial block, or 2^nd^/3^rd^ degree atrioventricular block), active peptic ulceration or gastrointestinal bleeding, severe hepatic or renal disease, malignancy within the past 5 years, or severely declined vision or hearing.
4. Subjects who had a history or presence of any contraindication for the application of AchEIs, a history of other concomitant neurodegenerative or psychiatric disease, a history of drug or alcohol addiction within the previous 10 years
5. Subjects who had severe ischemia based on the CREDOS WMH visual rating scale, and multiple large territorial infarctions or single strategically placed infarctions on MRI scan conducted at baseline or within the 12 months prior to the baseline examination.

**6. Intervention**

**6.1 Investigational drug**

Once a day, a patch size of 5 cm2 with a rivastigmine patch (containing 9 mg of rivastigmine and 4.5 mg delivered over 24 hours) and a once daily patch size of 10 cm2 rivastigmine patch 18 mg and 9.5 mg delivered over 24 hours) is administered as a therapeutic drug during the study period.

**6.2 Treatment regimen**

**6.2.1 Treatment schedule**

|  | Weeks 1-4 | Weeks 5-8 | Weeks 9-12 | Weeks 13-24 |
| --- | --- | --- | --- | --- |
| AD without white matter change | Patch 5 cm^2^ | Patch 10 cm^2^ | | |
|  |  | | |
| AD with white matter change | Patch 5 cm^2^ | Patch 10 cm^2^ | | |
|  | | | |

**6.2.2 Subject assignment and prescribing and administering medication**

Patient subjects will be allocated to AD without white matter change group or AD with white matter change group at the baseline. All patients start on a 5-cm^2^ patch and their dose is increased to a 10-cm^2^ patch after 4 weeks. White matter changes will be defined by CREDOS protocol. Periventricular white matter (PWM) changes are classified into P1 (caps or rim <5 mm), P2 (5 mm <caps or rim <10 mm), P3 (10 mm <caps or rim). Likewise, deep white matter (DWM) ischemic lesions are classified into D1 (the longest diameter of DWM lesion <10 mm), D2 (10mm<DWM<25mm), and D3 (25mm <DWM). The patients would be divided into two groups according to combinations of PWM and DWM ischemic lesions: Group I (without or minimal white matter changes: D1P1,) and Group II (mild to moderate white matter changes: D1P2, D2P1, D1-P3, D2-P2, D2-P3, D3-P1, D3-P2).

| **Treatment Arm** | **# of Patients Entered Treatment** | **Type of Study Drug** | **Compound** | **Min Dose** | **Max Dose** | **Frequency** | **Admin. Route** |
| --- | --- | --- | --- | --- | --- | --- | --- |
| AD without WMC | 200 | Exelon patch | Rivastgmine | 5cm^2^ | 10cm^2^ | qd | PO |
|  |  |  |  |  |  |
| AD with WMC | 100 | Exelon patch | Rivastgmine | 5cm^2^ | 10cm^2^ | qd | PO |
|  |  |  |  |  |  |

### 6.2.3 Controlling medication doses and suspending doses

If the subject is unable to tolerate the assigned dose, dose adjustment and suspension of dosing are allowed. The following treatment coordination schemes are recommended:

• In the case of a tolerability problem, the caregiver contacts the researcher as soon as possible.

• If the tolerability problem improves and subsequent days of treatment are less than 3 days, resume treatment to the same dose level as before administration. At this time, if tolerability is still a problem and the subject receives a patch of 5 cm², the subject may be dropped from the test. If tolerability is still a concern and the subject is treated with a 10 cm² patch, resume treatment at the lower dose level.

• If the tolerability problem improves and the days of consecutive administration are more than three days, resume treatment at a lower dose than before administration.

• If the subject's tolerability has improved, restart the dose titration schedule. If the tolerability problem is caused again at the next dose titration step, administration may be omitted again. Future attempts to titrate the dose will be at the discretion of the investigator (ie, if necessary for tolerance, the dosage does not necessarily have to be titrated to the target maximum dose).

• The dose level reduction required due to tolerability issues is permissible at any time during the treatment period.

### 6.2.4 Concomitant medication

All existing diseases or medical conditions are treated based on the prevailing medical practice.

The following treatments are not allowed after screening.

- All other clinical trial drugs
- Drugs or treatments known to cause major organ damage
- Rivastigmine capsule, donepezil, galantamine
- Muscle relaxant, general anesthesic medication
- Lithium
- Anticholinergic drugs (amantadine, atropine, benztropine, trihexyphenidyl)
- Tricyclic antidepressant
- Vitamine E, Estrogen, Gingko biloba, nootropics [ie. piracetam, Acetyl-L-carnitine, ergoloid mesylates, oxiracetam, Choline alphoscerate]

The following medications are allowed to be administered but with certain restrictions:

- - After screening, it is recommended that psychotropic drugs (antipsychotics, antidepressants, anxiolytics, anticonvulsants, sleeping pills) be discontinued or used with the minimum dose required for the patient's symptoms. If you cannot discontinue use, you can take part in this study if you are taking a mental agonist at a stable dose for a period of 4 weeks before baseline. Try to maintain this stable dose for the duration of the trial. All treatments initiated during the trial should be performed for the shortest possible period of time with the lowest effective dose.

The investigator will train the subject to inform the testing laboratory of all new drugs used since the start of the trial.

All drugs used after the subject started treatment with the test drug should be recorded in the Concomitant medications after start of study drug section of the CRF.

### 6.2.5 Discontinuation of trial drug treatment and early elimination of subjects

If the investigator believes that continuing the administration of the test drug will pose a significant safety risk to the subject, the subject should stop administering the test drug. In the following situations, the study medication may be discontinued according to the judgment of the investigator:

- If the investigator determines that treatment is discontinued due to abnormal or clinically significant changes in test results or abnormalities
- If the researcher determines that continuing treatment will be detrimental to the well-being of the subject

Subject to the following grounds, the subject is severely disqualified from the examination:

- Withdrawal of subject consent
- Stop taking the test drug for more than 8 days
- If the researcher determines that drop-out is best for the subject at any time for any reason

### A violation of the plan does not lead to the elimination of the subject unless the risk is significant to the safety of the subject. The subject may voluntarily cease to participate in the trial at any time for any reason. Subjects, who are willing to discontinue participation, do not want to visit, or fail to follow-up for any other reason may be considered dropouts.

### In the event of premature drop out, the investigator will assess the subject's primary cause of early withdrawal of the clinical trial and provide that information in the Study Completion section of the CRF. If the subject is unclear because he has not attended a clinical trial visit without expressing his intention to stop participating, the researcher should conduct a telephone conversation with the subject.

### 6.2.6 Termination of study

All subjects will receive a final final examination for efficacy and safety evaluation at the end of the 24-week treatment period. Subjects who discontinue treatment early after the baseline will be assessed at the end of the 24th week.

### 6.2.7 Early termination of study

Clinical trials may be terminated at any time at the discretion of the investigator meeting. If termination of the clinical trial is required, the subject will be interviewed and treated as soon as possible, as described for the 7 early withdrawal subjects. Additional procedures may be provided to the investigator to ensure that appropriate action is taken to protect the subject's interests. The investigator should report the early termination of the clinical trial to the IRB.

# 7 Study visit and assessment

We described all study visits schedule and assessment activities in table 1.

The subject will be present on all scheduled visits within 7 days from the specified date or 7 days prior to the specified date within 14 days. Subjects who have been dropped early in the test after the baseline for any reason will re-arrange their visit as soon as possible to perform all assessments scheduled at the final visit.

**Screening visit:** Subject / guardian consent, demographic and background information, inclusion and exclusion criteria, NINCDS-ADRDA 'probable' diagnostic criteria for Alzheimer's disease, current / past history, concurrent medication, vital signs, physical examination, height, weight, neurological examination, MMSE, and the screening confirmation.

**Baseline visit:** This visit will be conducted within 28 days of the screening visit.

The baseline assessment included concomitant medication, dose regimens, compliance, ADAS-cog, MMSE, FAB ADCS-ADL, CGA-NPI, CDR-SB, Mini-Zarit, vital sign, physical examination, .

**Treatment visits:** A safety assessment is carried out at each visit. Safety assessments include signs of vital signs, physical examination, weight, neurological examination, and adverse events. Therapeutic evaluations are performed at each visit and include concurrent medications, dose-related records. The efficacy assessment was performed at the end of 24 weeks and included ADAS-cog, MMSE, FAB, ADCS-ADL, CGA-NPI, CDR-SB and Mini-Zarit. Subjects who discontinue treatment early will be assessed at the end of treatment at the end of the 24th week.

**Unscheduled visits**: Perform an assessment related to the cause of the unscheduled visit.

Table 1. Study Process

| **Assessment and activities** | **Visit schedule** | | | | |
| --- | --- | --- | --- | --- | --- |
|  | **Visit 1** | **Visit 2** | **Visit 3** | **Visit 4** | **Visit 5** |
|  | **D-28~1** | **Day 0** | 4wk**±**7d | 12wk**±**7d | 24wk**±**7d, or  Early termination |
| Informed consent | X |  |  |  |  |
| Inclusion/Exclusion criteria | X |  |  |  |  |
| Medical history | X |  |  |  |  |
| NINCDS/ADRDA probable AD criteria | X |  |  |  |  |
| **Concomitant Medication** | | | | | |
| Concomitant medication | X | X | X | X | X |
| Drug dosage description | X | X |  | X |  |
| **Efficacy measure s** | | | | | |
| ADAS-cog (patient) |  | X |  |  | X |
| Caregiver burden(caregiver) |  | X |  |  | X |
| MMSE(patient) |  | X |  |  | X |
| ADCS-ADL (caregiver) |  | X |  |  | X |
| CGA-NPI (caregiver) |  | X |  |  | X |
| CDR-SB |  | X |  |  | X |
| **Safety measures** | | | | | |
| Vital sign | X | X |  | X | X |
| Heigt | X |  |  |  |  |
| Weight / Physical examination | X |  |  |  | X |
| Neurologic examination | X | X |  | X | X |
| Adverse events |  | X | X | X | X |
| Medication schedule | **screening** | **5cm^2^**  **patch** | **10cm^2^**  **patch** | | |

**7.1 Subject demographic and background information**

- Demographical data
- Vital sign: blood pressure,
- Weight, height
- Disease characteristics: NINCDS/ADRDA criteria for AD.
- Past and present medical history
- Family history and history for smoking and alcohol consumption

**7.2 Treatment exposure and compliance**

Enter the test drug dose prescribed to the subject in the Dosage Administration Record column with the start date, end date, dose, and reason for the dose adjustment.

Concomitant medications, including psychotropic drugs (antipsychotics, antidepressants, anxiolytics, anticonvulsants, sleeping pills) used in the treatment of emotions or behavior, are recorded in the CRF. Record the product name, start date, end date, and reason for use.

**7.3 Efficacy**

At the baseline and 24-week visits, the same evaluator takes charge of the same subject for the same examinations as possible.

- Alzheimer’s Disease Assessment Scale-Cognitive subscale (ADAS-cog)
- MMSE (Mini-Mental State Examination)
- Frontal Assessment Battery (FAB)
- Alzheimer’s Disease Cooperative Study - Activities of Daily Living (ADCS-ADL)
- Caregiver-Administered Neuropsychiatric Inventory (CGA-NPI)
- Clinical Dementia Rating Scale-Sum of Boxes (CDR-SB)
- Caregiver burden scale Korean version of mini-Zarit

**7.4 Safety**

### 7.4.1 Physical and neurologic examination

Physical examinations include examination of the general appearance, skin, neck (including thyroid), eyes (except for fundus examination), ears, nose, throat, lung, heart, abdomen, back, lymph nodes, and limbs. Neurological examinations include cranial nerves, limb movements and sensory functions, deep key streaks, Babinski reflexes, Chaddock signs, and cerebellar function tests.

**7.4.2 Height, weight, vital signs**

- Height: Evaluate only at screening. Measure in centimeters (cm).
- Weight: Weighs up to 0.1 kilograms (kg) with naked robes and shoes on each visit.
- Vital sign:

After resting for 5 minutes, use a mechanical sphygmomanometer to measure pulse, systolic and diastolic blood pressure in sitting position.

## 7.4.3 Adverse events

An adverse event means the appearance or aggravation of any undesirable signs, symptoms, or medical conditions that occur after obtaining the subject consent, regardless of the test drug relevance. Medical conditions / illnesses that existed prior to obtaining the subject consent are considered adverse events only if they have deteriorated since the start of the study. Abnormal laboratory performance figures and test results are adverse events only if they cause clinical signs or symptoms, cause the test drug to stop, or require treatment.

The occurrence of adverse events should be investigated by questioning each subject at each visit during the clinical trial. Adverse events may also be identified by voluntary reporting by the subject during visits or during visits, or by physical examination, laboratory testing, or other evaluation. For all adverse events, record the following information in the Adverse Events section of the CRF:

1. Severity (mild, moderate, severe)

2. Causal relationship with test drug (estimated / unspecified)

3. Duration (start date and end date, or duration of last test)

4. Is it a serious adverse events (SAE)

**A serious adverse events (SAE) is defined as:**

• Lethal or life-threatening

• Continuous or significant disability / impairment of function

• Congenital defects or birth defects

• In a medical emergency, i.e. when the subject is at risk or needs medical or surgical intervention to prevent the consequences described above.

• Admission is not considered a serious adverse event if hospitalization or extension of existing hospitalization is required, such as:

- Common treatments not related to disease worsening

- Selective and pre-planned treatment of existing disease that is not related to the indications of this trial and has not deteriorated since the beginning of the study drug

- Emergency outpatient treatment that does not fall within the definition of serious adverse events described above and does not result in hospitalization

- Societal reasons and deterioration of general condition of the patient are not accompanied by short-term hospital admission

Unlike routine safety assessments, critical adverse events are continuously monitored and reported in accordance with specific reporting procedures (see section 7.5).

All adverse events should be treated appropriately. Such treatment may include one or more of the following measures: no action taken (i.e., continuous observation), test drug dose adjustment / intermittent discontinuation; Permanent discontinuation of the test drug due to the adverse event; Concomitant drug administration; Enforcement of non-medication laws; Subject Admission / Extension of the period of stay for the subject. Record the action taken to treat the adverse event in the Adverse Event section of the CRF.

If an adverse reaction is found, follow-up should be done until the adverse event is resolved or determined to be permanent, and the change in severity at each visit, its relevance to the test drug.

## 7.5 Report of serious adverse events

To ensure patient safety, every SAE, regardless of suspected causality, occurring after the patient signs the informed consent and until 4 weeks after the patient has stopped study treatment must be reported to Novartis Safety Desk within 15 days of learning of its occurrence. Any SAEs experienced after this 4-week period should only be reported to Novartis Safety Desk if the investigator suspects a causal relationship to the study drug.

Recurrent episodes, complications, or progression of the initial SAE must be reported as follow-up to the original episode within 15 days of the investigator receiving the follow-up information. An SAE occurring at a different time interval or otherwise considered completely unrelated to a previously reported one should be reported separately as a new event.

Information about all SAEs is collected and recorded on the Serious Adverse Event Report Form. The investigator must assess and record the relationship of each SAE to each specific study drug (if there is more than one study drug), complete the SAE Report Form in English, and send the completed, signed form within 15 days to Novartis Safety Desk.

**7.6 Report of pregnancy**

To ensure patient safety, each pregnancy in a patient on study drug must be reported to Novartis. The pregnancy should be followed up to determine outcome, including spontaneous or voluntary termination, details of the birth, and the presence or absence of any birth defects, congenital abnormalities, or maternal and/or newborn complications.

Pregnancy should be recorded on a Clinical Trial Pregnancy Form and reported by the investigator to the local Novartis Safety Desk according to ‘SAE/IN, Pregnancy Reporting Procedures’. Pregnancy follow-up should be recorded on the same form and should include an assessment of the possible relationship to the Novartis study drug of any pregnancy outcome. Any SAE experienced during pregnancy must be reported on the SAE Report Form.


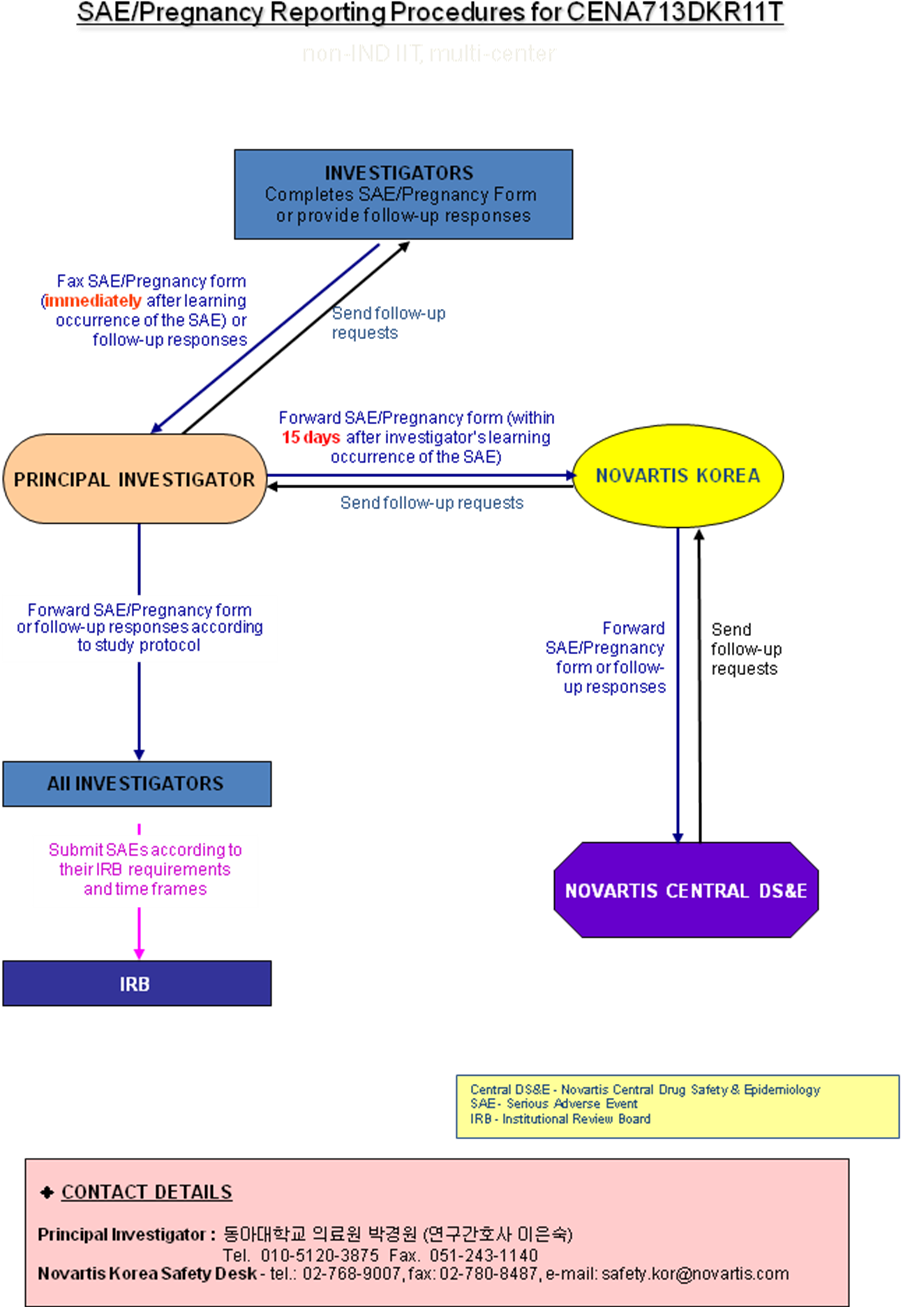


# 8. Data analysis

## 8.1 Analysis group

Data were summarized using descriptive statistics: frequency and percentage for categorical variables and mean ± SD for continuous variables. Differences in study participants' characteristics were compared across subgroups using chi-square tests or Fisher’s exact tests for categorical variables and independent t tests or Mann-Whitney’s U tests for continuous variables, as appropriate. To check for normal distribution, we used the Shapiro-Wilk’s test. Changes at end point from baseline were compared using an analysis of covariance (ANCOVA) model, with the use of the baseline score as a covariate. We used bar charts for data visualization. Intent to treat (ITT) and per protocol (PP) analyses were performed and the last observed carried forward (LOCF) method was used to impute missing values. P values lower than 0.05 were considered statistically significant. All statistical analyses were carried out using Statistical Package for Social Sciences 22.0 (SPSS Statistics for Windows 22.0, Armonk, NY, IBM Corp.) statistical software. All tests were two-tailed.

## 8.2 Subject demographic and background information

Data were summarized using descriptive statistics: frequency and percentage for categorical variables and mean ± SD for continuous variables. Differences in study participants' characteristics were compared across subgroups using chi-square tests or Fisher’s exact tests for categorical variables and independent t tests or Mann-Whitney’s U tests for continuous variables, as appropriate.

**8.3 Analysis of primary objective**

### 8.3.1 Evaluation variables statistical hypothesis, model and method analysis

Efficacy measures were assessed at baseline and on week 24. Primary outcome was the change from baseline on the Alzheimer’s Disease Assessment Scale-Cognitive subscale on week 24 (ADAS-Cog).

## Analysis of secondary objective

Secondary outcomes were as follows: MMSE, Clinical Dementia Rating Scale-Sum of Boxes (CDR-SB) [26], Frontal Assessment Battery (FAB) [27], Caregiver-Administered Neuropsychiatric Inventory (CGA-NPI) [28], Alzheimer’s Disease Cooperative Study-Activities of Daily Living (ADCS-ADL) [29], and Caregiver Burden Scale-Korean Version of Mini-Zarit [30]. Safety evaluations, including vital signs, neurological examinations, and adverse event (AE) and serious AE (SAE) monitoring were performed regularly throughout the study.

**9. Ethical consideration**

## Compliance with related laws and ethical principles

The study was performed in accordance with the International Harmonization Conference guidelines on Good Clinical Practice and was approved by the institutional review board of each center prior to the beginning the study.

## Subject consent procedure

Prior to participation in the study, all participants or their legally authorized representatives provided written informed consent to participate in the study.

# Compliance with clinical trial plan

The study was performed in accordance with the International Harmonization Conference guidelines on Good Clinical Practice.

## Changes in clinical trial plan

The study protocol changes should be approved by the institutional review board of each center prior to the process of the study.

**10. Reference**

Yamada M, Mimori Y, Kasagi F, Miyachi T, Ohshita T, Sudoh S, et al. Incidence of dementia, Alzheimer disease, and vascular dementia in a Japanese population: Radiation Effects Research Foundation adult health study. Neuroepidemiology. 2008;30(3):152-60.

Whitehouse PJ, Price DL, Clark AW, Coyle JT, DeLong MR. Alzheimer disease: evidence for selective loss of cholinergic neurons in the nucleus basalis. Ann Neurol. 1981 Aug;10(2):122-6.

Rogers SL, Farlow MR, Doody RS, Mohs R, Friedhoff LT. A 24-week, double-blind, placebo-controlled trial of donepezil in patients with Alzheimer's disease. Donepezil Study Group. Neurology. 1998 Jan;50(1):136-45.

Tariot PN, Solomon PR, Morris JC, Kershaw P, Lilienfeld S, Ding C. A 5-month, randomized, placebo-controlled trial of galantamine in AD. The Galantamine USA-10 Study Group. Neurology. 2000 Jun;54(12):2269-76.

Rösler M, Anand R, Cicin-Sain A, Gauthier S, Agid Y, Dal-Bianco P, et al. Efficacy and safety of rivastigmine in patients with Alzheimer's disease: international randomised controlled trial. BMJ. 1999 Mar;318(7184):633-8.

Erkinjuntti T. Vascular dementia: challenge of clinical diagnosis. Int Psychogeriatr. 1997;9 Suppl 1:51-8; discussion 77-83.

Heyman A, Fillenbaum GG, Welsh-Bohmer KA, Gearing M, Mirra SS, Mohs RC, et al. Cerebral infarcts in patients with autopsy-proven Alzheimer's disease: CERAD, part XVIII. Consortium to Establish a Registry for Alzheimer's Disease. Neurology. 1998 Jul;51(1):159-62.

Sakurada T, Alufuzoff I, Winblad B, Nordberg A. Substance P-like immunoreactivity, choline acetyltransferase activity and cholinergic muscarinic receptors in Alzheimer's disease and multi-infarct dementia. Brain Res. 1990 Jun;521(1-2):329-32.

Tohgi H, Abe T, Kimura M, Saheki M, Takahashi S. Cerebrospinal fluid acetylcholine and choline in vascular dementia of Binswanger and multiple small infarct types as compared with Alzheimer-type dementia. J Neural Transm. 1996;103(10):1211-20.

Mesulam M, Siddique T, Cohen B. Cholinergic denervation in a pure multi-infarct state: observations on CADASIL. Neurology. 2003 Apr;60(7):1183-5.

Swartz RH, Sahlas DJ, Black SE. Strategic involvement of cholinergic pathways and executive dysfunction: Does location of white matter signal hyperintensities matter? J Stroke Cerebrovasc Dis. 2003 Jan;12(1):29-36.

Pratt RD, Perdomo CA. Donepezil-treated patients with probable vascular dementia demonstrate cognitive benefits. Ann N Y Acad Sci. 2002 Nov;977:513-22.

Black S, Román GC, Geldmacher DS, Salloway S, Hecker J, Burns A, et al. Efficacy and tolerability of donepezil in vascular dementia: positive results of a 24-week, multicenter, international, randomized, placebo-controlled clinical trial. Stroke. 2003 Oct;34(10):2323-30.

Wilkinson D, Doody R, Helme R, Taubman K, Mintzer J, Kertesz A, et al. Donepezil in vascular dementia: a randomized, placebo-controlled study. Neurology. 2003 Aug;61(4):479-86.

Erkinjuntti T, Kurz A, Gauthier S, Bullock R, Lilienfeld S, Damaraju CV. Efficacy of galantamine in probable vascular dementia and Alzheimer's disease combined with cerebrovascular disease: a randomised trial. Lancet. 2002 Apr;359(9314):1283-90.

Orgogozo JM, Rigaud AS, Stöffler A, Möbius HJ, Forette F. Efficacy and safety of memantine in patients with mild to moderate vascular dementia: a randomized, placebo-controlled trial (MMM 300). Stroke. 2002 Jul;33(7):1834-9.

Wilcock G, Möbius HJ, Stöffler A, group M. A double-blind, placebo-controlled multicentre study of memantine in mild to moderate vascular dementia (MMM500). Int Clin Psychopharmacol. 2002 Nov;17(6):297-305.

Moretti R, Torre P, Antonello RM, Cazzato G. Rivastigmine in subcortical vascular dementia: a comparison trial on efficacy and tolerability for 12 months follow-up. Eur J Neurol. 2001 Jul;8(4):361-2.

Moretti R, Torre P, Antonello RM, Cazzato G, Bava A. Rivastigmine in subcortical vascular dementia: an open 22-month study. J Neurol Sci. 2002 Nov;203-204:141-6.

Ballard C, Sauter M, Scheltens P, He Y, Barkhof F, van Straaten EC, et al. Efficacy, safety and tolerability of rivastigmine capsules in patients with probable vascular dementia: the VantagE study. Curr Med Res Opin. 2008 Sep;24(9):2561-74.

Tsujimoto S, Sakaki T, Morimoto T, Tominaga M. The effect of acetylcholinesterase inhibitor (SDZ ENA 713) for r-CBF and focal cerebral ischaemia. Acta Neurochir (Wien). 1993;124(2-4):127-31.

Kumar V, Anand R, Messina J, Hartman R, Veach J. An efficacy and safety analysis of Exelon in Alzheimer's disease patients with concurrent vascular risk factors. Eur J Neurol. 2000 Mar;7(2):159-69.

Winblad B, Cummings J, Andreasen N, Grossberg G, Onofrj M, Sadowsky C, et al. A six-month double-blind, randomized, placebo-controlled study of a transdermal patch in Alzheimer's disease--rivastigmine patch versus capsule. Int J Geriatr Psychiatry. 2007 May;22(5):456-67.

Oertel W, Ross JS, Eggert K, Adler G. Rationale for transdermal drug administration in Alzheimer disease. Neurology. 2007 Jul;69(4 Suppl 1):S4-9.

Dubois B, Slachevsky A, Litvan I, Pillon B. The FAB. A Frontal Assessment Battery at bedside. Neurology 2000;55:1621-1626.
