# Supplementary material for: Efficacy and tolerability of rivastigmine patch therapy in patients with mild-to-moderate Alzheimer’s dementia associated with minimal and moderate ischemic white matter hyperintensities: A multicenter prospective open-label clinical trial
Source: PLoS One. 2017 Aug 7;12(8):e0182123. doi: 10.1371/journal.pone.0182123 (PMC5546604; doi:10.1371/journal.pone.0182123)
Supplement: S1 Checklist — (PDF) [file pone.0182123.s003.pdf]

TREND Statement Checklist

Paper Section/ Topic	Item No	Descriptor	Reported?	
			✓	Pg #
**Title and Abstract**				
Title and Abstract	1	• Information on how unit were allocated to interventions	✓	1
		• Structured abstract recommended	✓	2
		• Information on target population or study sample	✓	2
**Introduction**				
Background	2	• Scientific background and explanation of rationale	✓	5
		• Theories used in designing behavioral interventions		
**Methods**				
Participants	3	• Eligibility criteria for participants, including criteria at different levels in recruitment/sampling plan (e.g., cities, clinics, subjects)	✓	7
		• Method of recruitment (e.g., referral, self-selection), including the sampling method if a systematic sampling plan was implemented	✓	7
		• Recruitment setting	✓	8
		• Settings and locations where the data were collected	✓	8
Interventions	4	• Details of the interventions intended for each study condition and how and when they were actually administered, specifically including:	✓	8
		o Content: what was given?	✓	9
		o Delivery method: how was the content given?	✓	9
		o Unit of delivery: how were the subjects grouped during delivery?	✓	8
		o Deliverer: who delivered the intervention?	✓	9
		o Setting: where was the intervention delivered?	✓	9
		o Exposure quantity and duration: how many sessions or episodes or events were intended to be delivered? How long were they intended to last?	✓	9
		o Time span: how long was it intended to take to deliver the intervention to each unit?	✓	9
		o Activities to increase compliance or adherence (e.g., incentives)		
Objectives	5	• Specific objectives and hypotheses	✓	6
Outcomes	6	• Clearly defined primary and secondary outcome measures	✓	9
		• Methods used to collect data and any methods used to enhance the quality of measurements	✓	9
		• Information on validated instruments such as psychometric and biometric properties	✓	5
Sample Size	7	• How sample size was determined and, when applicable, explanation of any interim analyses and stopping rules	✓	9
Assignment Method	8	• Unit of assignment (the unit being assigned to study condition, e.g., individual, group, community)	✓	8
		• Method used to assign units to study conditions, including details of any restriction (e.g., blocking, stratification, minimization)	✓	8
		• Inclusion of aspects employed to help minimize potential bias induced due to non-randomization (e.g., matching)		
